# Chemical and Biological Research on Herbal Medicines Rich in Xanthones

**DOI:** 10.3390/molecules22101698

**Published:** 2017-10-11

**Authors:** Jingya Ruan, Chang Zheng, Yanxia Liu, Lu Qu, Haiyang Yu, Lifeng Han, Yi Zhang, Tao Wang

**Affiliations:** 1 Tianjin State Key Laboratory of Modern Chinese Medicine, 312 Anshanxi Road, Nankai District, Tianjin 300193, China; Ruanjy19930919@163.com (J.R.); 18702270347@163.com (C.Z.); liuyanxia210@163.com (Y.L.); 2 Tianjin Key Laboratory of TCM Chemistry and Analysis, Institute of Traditional Chinese Medicine, Tianjin University of Traditional Chinese Medicine, 312 Anshan Road, Nankai District, Tianjin 300193, China; qululuhan88@163.com (L.Q.); yuhaiyang19830116@hotmail.com (H.Y.); hanlifeng_1@163.com (L.H.)

**Keywords:** herbal medicines, xanthones, plant sources, pharmacology, gambogic acid, structure-activity relationships

## Abstract

Xanthones, as some of the most active components and widely distributed in various herb medicines, have drawn more and more attention in recent years. So far, 168 species of herbal plants belong to 58 genera, 24 families have been reported to contain xanthones. Among them, *Calophyllum*, *Cratoxylum*, *Cudrania*, *Garcinia*, *Gentiana*, *Hypericum* and *Swertia* genera are plant resources with great development prospect. This paper summarizes the plant resources, bioactivity and the structure-activity relationships (SARs) of xanthones from references published over the last few decades, which may be useful for new drug research and development on xanthones.

## 1. Introdution

Xanthones (IUPAC name 9*H*-xanthen-9-one) are a kind of phenolic acid with a three-ring skeleton, widely distributed in herbal medicines. These constituents display a vast range of bioactitivies, including anticancer, anti-oxidative, antimicrobial, antidiabetic, antiviral, and anti-inflammatory effects. So far, at least 515 natural xanthones from 20 families of higher plants (122 species in 44 genera) have been summarized in a few reviews [1,2,3]. These reviews were limited to xanthones with anticancer and anti-inflammatory activities [4]. Their structure-activity relationships (SARs) were also not mentioned.

Over the past few decades, xanthones have become an important resource for drug development. For example, gambogic acid, a prenyl xanthone isolated from *Garcinia hanburyi* (Clusiaceae), exhibited remarkable apoptosis, cell proliferation and tumor angiogenesis bioactivities, along with anti-oxidant, and anti-inflammatory activities [5,6] and synergistic anticancer activity [7,8]. A phase II clinical trial using gambogic acid in combination with anticancer drugs was carried out in China [9]. Besides gambogic acid mentioned above, mangosteen, another of the most well-known xanthones, has been used as a dietary supplement to improve immune function, decrease serum C-reactive protein levels and increase the ratio of T helper cells [10].

Xanthones are mainly isolated from herbal medicines. Between 1988 and 2016, 168 species of herbal medicinal plant belonging to 58 genera, and 24 families were reported to contain xanthones. This review summarizes the phytochemistry, bioactivity and structure-activity relationships (SARs) of xanthones, which may be helpful to the further new drug research and development.

## 2. Plant Sources of Xanthones

Table 1 summarizes the phytochemical research on xanthones found in 168 plant species belonging to 58 genera and 24 families.

Among them, the Calophyllaceae, Gentianaceae and Guttiferae are the most widely distributed families.

## 3. Bioactivities of Xanthones

Recently, some xanthones have been reported to be useful in the treatment of cancer, oxidation, microbial infection, diabetes, inflammation, virus infection et al. Target-based and structure-based activity evaluation has revealed that xanthones are good source of medicine for the treatment of various type of disease. In this part, we summarized pharmacological activities and SARs result of xanthones.

### 3.1. Effects on Cytotoxicity and Proliferation

**Figure 1:**
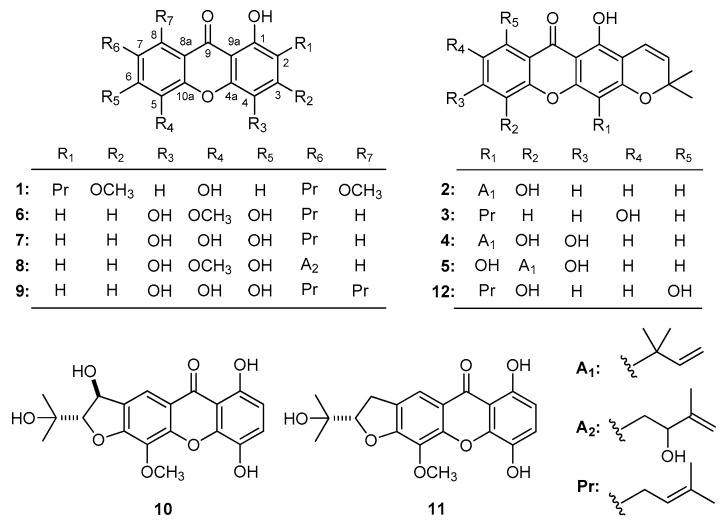
The structures of compounds **1**–**12**.

Cancer cytotoxicities of xanthones against leukemia cell lines were evaluated, which were isolated from *Artocarpus* [154], *Calophyllum* [34], *Garcinia* [178], *Hypericum* [140] genera herbal medicines. On the other hand, the xanthones phylattrin (**1**), caloxanthone C (**2**), brasixanthone B (**3**), macluraxanthone (**4**), and soulattrin (**5**) (Figure 1) obtained from *C. soulattri* [34] showed cytotoxic activities against the chronic myelogenous leukemia cell line (K562) (IC_50_: 22.10 ± 0.61, 18.20 ± 0.76, 31.00 ± 0.21, 5.28 ± 0.22, and 2.23 ± 0.13 μM, respectively). Their MTT test results indicated that with increasing number of hydroxyl groups, the anti-proliferative activity was enhanced (**2** < **4**).

In addition, the tests carried out by Niu et al. [43] supplemented the conclusions mentioned above. Thirty-one kinds of xanthones, including 1,4,6-trihydroxy-5-methoxy-7-prenylxanthone (**6**), 1,4,5,6-tetrahydroxy-7-prenylxanthone (**7**), bracteaxanthone III (**8**), 1,4,5,6-tetrahydroxy-7,8-di(3-methylbut-2-enyl)xanthone (**9**), bracteaxanthones V (**10**), IV (**11**), and garcinexanthone B (**12**) (Figure 1) were obtained from *G. bracteata*. Activity screening results revealed that the isoprenyl group played an important role in the HL-60 cytotoxicity. Among them, **7** and **9** showed stronger inhibitory abilities with IC_50_ at 10.1 ± 3.1, 2.8 ± 1.1 μM, respectively. The activities difference between these compounds suggested that along with the number of isoprenyl group increasing, the cytotoxicities became stronger. Meanwhile, the hydroxylation (**6** > **8**) or the cyclization into a furan or pyran ring of isoprenyl group (**6** > **10**–**12**) lowered the activity compared with corresponding compounds (IC_50_: 9.9 ± 0.8, 21.0 ± 0.5, 22.2 ± 0. 6, 18.0 ± 0.7 and 22.8 ± 0.4 μM for **6**, **8**, **10**, **11** and **12**, respectively).

**Figure 2:**
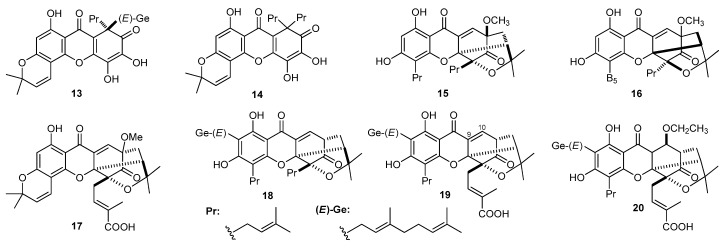
The structures of compounds **13**–**20**.

Xanthones from *Garcinia* [66], *Polygala* [166] genera plants were found to exhibit inhibitory activities in A549 lung cancer cell line. Oliganthins H (**13**), I (**14**), gaudichaudione H (**15**), cantleyanone (**16**), and oliganthone B (**17**) (Figure 2) isolated from *G. oligantha* [66] showed anti-proliferative potency for A549 with IC_50_ at 5.0 ± 0.32, 5.5 ± 0.47, 3.0 ± 0.49, 2.9 ± 0.42, 3.9 ± 0.86 μM, respectively. Compounds **15, 16** and **17** exhibited stronger inhibitory abilities compared with **13** and **14**, which indicated that caged-xanthones may have better performances on inhibiting the growth of A549 cell line. A cytotoxicity screen on desoxygambogenin (**18**), isogambogenic acid (**19**), and 10α-ethoxy-9,10-dihydrogambogenic acid (**20**) (Figure 2) from *G. hanburyi* [53] against the A549 cell line indicated that the carboxylation of the side chain of the caged-xanthones decreased the activity (**18** > **19**), and the caged-xanthones with an olefinic bond between C-9 and C-10 depslayed higher inhibitory ability than hydroxyl substituted ones (**19** > **20**).

**Figure 3:**
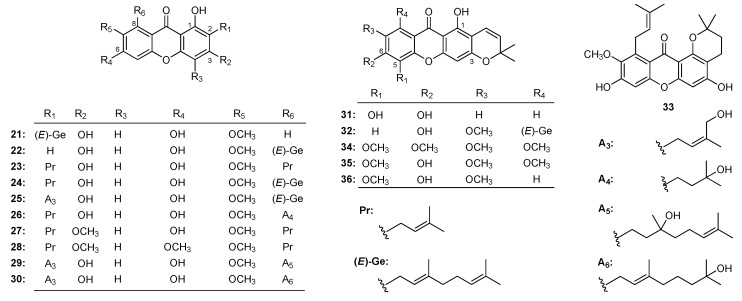
The structures of compounds **21**–**36**.

Xanthones isolated from *Garcinia* genus plants showed significant inhibitory effect in colon cancer cells. The cytotoxicity of cowaxanthone (**21**), rubraxanthone (**22**), α-mangostin (**23**), cowanin (**24**), cowanol (**25**) (Figure 3) from *G. oliveri* [67] against DLD-1 cell line was confirmed by the MTT method with IC_50_ value at 24.4 ± 0.7, 33.9 ± 2.2, 12.2 ± 0.4, 13.2 ± 0.2, 14.8 ± 2.1 μM, respectively. SARs results suggested that free 3,6-dihydroxyl group and isoprenyl side chain at C-2 and C-8 of were active units.

α-Mangostin (**23**), cowanin (**24**), cowanol **(25)**, garcinone D (**26**), β-mangostin (**27**), fuscaxanthone C (**28**), fuscaxanthone I (**29**), kaennacowanol A (**30**), jacareubin (**31**), fuscaxanthone A (**32**), and 1-isomagostin (**33**) (Figure 3) were obtained from *G. cowa* [179]. To cervical cancer Hela cell line, IC_50_ value of cytotoxicities were 13.69, 11.68, 12.19, 22.58, 12.78, inactive, 17.20, 16.70, 11.43, inactive, 34.04 μM, respectively. SARs analysis results indicated that geranyl moiety at C-8 (**24**, **25** > **23**; **29**, **30** > **26**) and the hydroxyl group at C-1, C-3, C-5 and C-6 enhanced their cytotoxicities (**23** > **33**, **24** > **32**, **31** > **32**, **27** > **28**).

Cylindroxanthones A–C (**34**, **35** and **36**) (Figure 3) were gained from *G. cylindrocarpa* [180], along with the increase of methoxy, the anti-proliferative potency against oral epidermoid KB cell line (IC_50_: 2.36, 59.05 and 57.24 μM for **34**, **35** and **36** respectively) increased. The results indicated that the oxidation of the unsaturated isoprenyl group reduced anti-proliferative activity.

**Figure 4:**
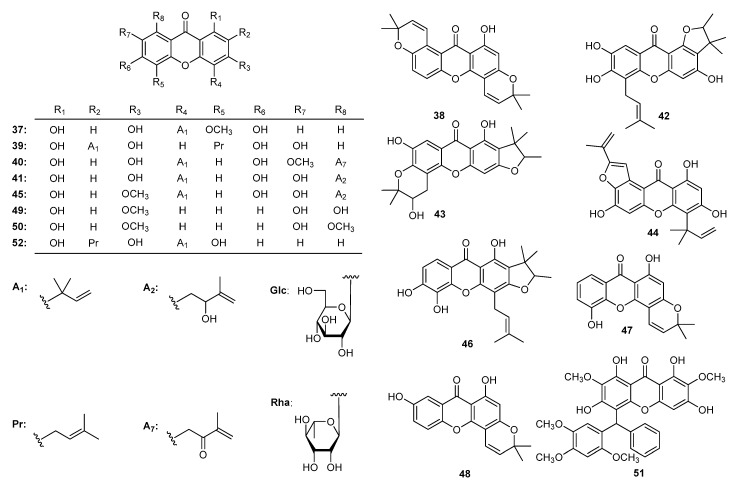
The structures of compounds **37**–**52**.

Neuroblastoma (SHSY5Y) cell line proliferation could be inhibited by neriifolone A (**37**), cudraxanthones A (**38**), L (**39**), cudratrixanthones C (**40**), G (**41**), H (**42**), I (**43**), O (**44**), 3-*O*-methyl-cudratrixanthone G (**45**), gerontoxanthone C (**46**), 6-deoxyisojacareubin (**47**), and nigrolineaxanthone F (**48**) (Figure 4) [181]. Moreover, gentiakochianin (**49**) and gentiacaulein (**50**) from *G. kochiana* [106] were also proved to promote cell cycle arrest in G2/M and G0/G1 phases in U251 human glioma cell line. Muchimangin B (**51**) [172] and allanxanthone A (**52**) (Figure 4) [23] inhibited the growth of pancreatic cancer (PANC-1), multiple myeloma (RPMI8226) and gastric cancer (BGC-823) cell lines. The SARs of above xanthones were not discussed for limitation on test sample number.

Although some xanthones showed significant inhibitory effects on cancer cell growth in vitro, the in vivo validatation report is rare, which limited their potential for development into new drug for anticancer.

### 3.2. Free Radical Scavenging Activity

Free radicals are defined as atoms with one unpaired electron, which can be formed through natural physiological processes. Overproduction of free radicals can accelerate the progression of cancer, cardiovascular disease, and age-related diseases.

**Figure 5:**
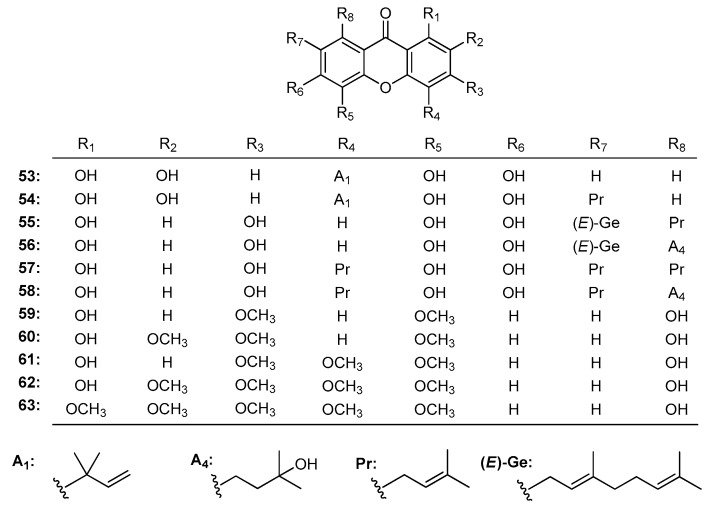
The structures of compounds **53**–**63**.

Symphoxanthone (**53**), subeliptenone B (**54**), garciniaxanthone E (**55**), garcinenone D (**56**), 1,3,5,6-tetrahydroxy-4,7,8-tri(3-methyl-2-butenyl)xanthone (**57**), garcinenone E (**58**) (Figure 5) were isolated from *G. xanthochymus* [84]. Their IC_50_ values against 1,1-diphenyl-2-picrylhydrazyl (DPPH) radical were 6.4, 6.0, 10.1, 6.8, 10.1, 8.5 μM, respectively. SARs analysis indicated that the radical-scavenging activity was partly related to phenolic hydroxy moiety numbers. Within them, compounds with *ortho* diphenolic hydroxy groups showed significant radical-scavenging activity.

As described above, 1,8-dihydroxy-4,6-dimethoxyxanthone (59), 1,8-dihydroxy-4,6,7-trimethoxyxanthone (**60**), 1,8-dihydroxy-4,5,6-trimetoxyxanthone (**61**), 1,8-dihydroxy-4,5,6,7-tetra-methoxyxanthone (**62**), 1-hydroxy-4,5,6,7,8-pentamethoxyxanthone (**63**) (Figure 5) [162]. The DPPH scavenging order was **59** (1.3 μg) < **63** (0.6 μg) < **60** and **61** (0.3 μg) < **62** (0.15 μg). Agreeing with literature reports, the results indicated that DPPH radical scavenging activities might be attributed to the phenol-like OH groups at the xanthone skeleton [182].

### 3.3. Anti-Microbial Activity

Xanthones show suppressive effects on microorganisms, such as Gram-positive or negative bacteria and fungi. The resources include *Allanblackia* [23], *Cassia* [102], *Centaurium* [106], *Cratoxylum* [40], *Garcinia* [183], *Hypericum* [97], *Kielmeyera* [87], *Psorospermum* [91], *Swertia* [133], *Usnea* [160], and *Vismia* [96] genera plants.

**Figure 6:**
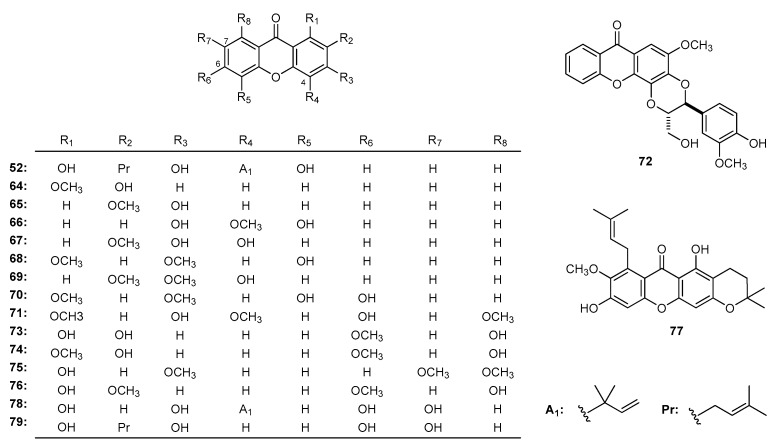
The structures of compounds **52**, **64**–**79**.

2-Hydroxy-1-methoxyxanthone (**64**), 3-hydroxy-2-methoxyxanthone (**65**), 3,5-dihydroxy-4-methoxyxanthone (**66**), 3,4-dihydroxy-2-methoxyxanthone (**67**), 5-hydroxy-1,3-dimethoxyxanthone (**68**), 4-hydroxy-2,3-dimethoxyxanthone (**69**), 3,4-dihydroxy-6,8-dimethoxyxanthone (**70**), 3,6-di-hydroxy-1,4,8-trimethoxyxanthone (**71**), and kielcorin (**72**) (Figure 6) obtained from *K. variabilis* [87] showed strong activities against EMRSA-16. According to the results, phenol-like OH groups at the xanthone skeleton may play an important role in the inhibitory ability on the proliferation of microorganisms (MIC: 32, 32, 32, 16, 64, 64, > 512 mg/L for **64**, **65**, **66** + **70** + **71**, **67**, **68**, **69**, **72**, respectively). The isolates 1,7,8-trihydroxy-3-methoxyxanthone (**73**), gentiacaulein (**74**), and decussatin (**75**) (Figure 6) obtained from *S. mussotii* [133] were proved to inhibit the growth of *M. tuberculosis* with the same MICs at 125 μg/mL, while 1,8-dihydroxy-2,6-dimethoxyxanthone (**76**) exhibited negative results. The SAR analysis indicated that the C-2, 4, 5 hydroxyl or methoxyl on the xanthone skeleton may influence the activities in resisting bacterial infection.

The antibacterial capacity of α-mangostin (**23**), cowanin (**24**), fuscaxanthone A (**32**), 9-hydroxycalabaxanthone (**77**) (Figure 6) [183] against *Staphylococcus aureus* suggested that the increase of the unsaturated isoprenyl groups lowered the anti-bacterial ability. Boonnak et al. [40] reported that 1,3,7-trihydroxyxanthones with isoprenyl or geranyl side chain and 1,3,7-trioxygenated xanthone with geranyl side chain showed strong inhibitory activity on *P. aeruginosa* (a kind of Gram-negative bacteria).

Allanxanthones A (**52**), D (**78**) and 1,3,6,7-tetrahydroxy-2-(3-methylbut-2-enyl)xanthone (**79**) (Figure 6) obtained from the stem bark of *A. gabonensis* [23] were studied for their antifungal ability against *Candida krusei*. Allanxanthone D (**78**) showed stronger antibiotic activity than the reference antibiotic (IC_50_ μg/mL: 1.22, 2.44, 2.44, 4.88, for **52**, **78**, **79**, and the reference antibiotic nystatin, respectively), which indicated that the oxygen substitution at C-6, 7 and the prenyl substitution at C-4 may be the active units.

### 3.4. α-Glucosidase Inhibitory Activity

**Figure 7:**
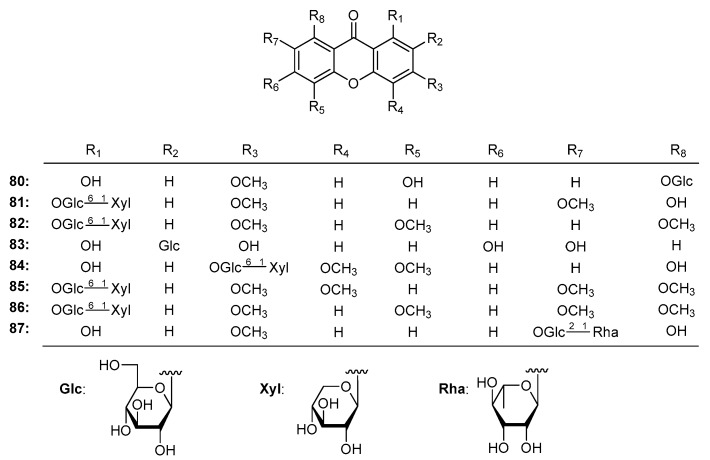
The structures of compounds **80**–**87**.

Plants resources with α-glucosidase inhibitory activity include the *Cudrania* [155], *Garcinia* [184], and *Swertia* [130] genera. Swertianolin (**80**), 1-*O*-[β-d-xylopyranosyl-(1→6)-β-d-glucopyranosyl]-8-hydroxy-3,7-dimethoxyxanthone (**81**), kouitchenside D (**82**), mangiferin (**83**), kouitchensides F (**84**), B (**85**), E (**86**), kouitchenside I (**87**) (Figure 7) isolated from *S. kouitchensis* [130] showed inhibitory effects on α-glucosidase with IC_50_ values of 126 ± 23, 451 ± 41, 360 ± 39, 296 ± 52, 184 ± 23, 383 ± 18 and 371 ± 22 μM, inactive, respectively. The results revealed that the substitution with a primeverosyl residue led to increased inhibitory effects than other diglycoside units (**81**, **82**, **84**, **85**, and **86** > **87**), and oxygen substitution at C-1 or C-8 (**80**, **83** and **84**), while a diglycoside residue located at C-7 (**87**) reduced the inhibitory activity.

### 3.5. Anti-Virus Activity

**Figure 8:**
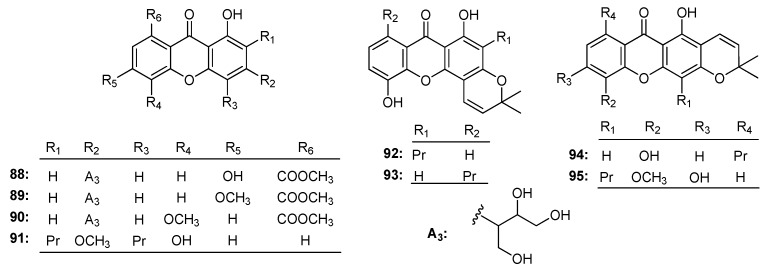
The structures of compounds **88**–**95**.

Xanthones obtained from *Comastoma* [108], *Garcinia* [178], and *Swertia* [128] genera exhibited inhibitory effects on tobacco mosaic virus (TMV). The anti-TMV half-leaf tests on paucinervins E (**88**), F (**89**), G (**90**), cudraxanthone G (**91**), ananixanthone (**92**), merguenone (**93**), nigrolineaxanthone K (**94**), 5-*O*-methylxanthone V1 (**95**) (Figure 8) (IC_50_: 21.4 ± 2.3, 42.8 ± 3.0, 53.6 ± 2.2, 52.8 ± 3.0, 68.9 ± 2.3, 82.4 ± 2.6 μM, for **88**, **89**, **90**, **91**, **92**, **95**, respectively) [107] suggested that the hydroxyl groups might be one of the active units (**88** > **89** and **90**). The introduction of the pyran ring (**92**, **93**, **94**, and **95**) or the interaction through hydrogen bonding with an isoprenyl group (**91**) would lower the inhibitory activities.

### 3.6. Anti-Inflammatory Activity

**Figure 9:**
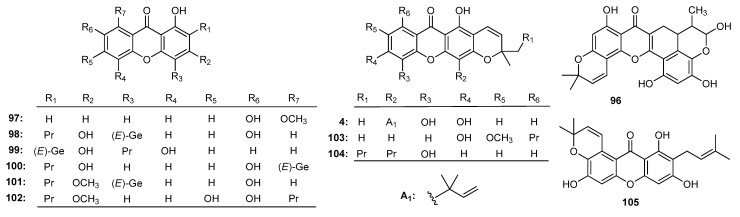
The structures of compounds **4**, **96**–**105**.

During the past ten years, plants belonging to the *Artocarpus* [154], *Calophyllum* [185], *Cratoxylum* [41], and *Garcinia* [46] genera have been reported to display anti-inflammatory activity. Pyranocycloartobiloxanthone A (**96**, Figure 9), a novel xanthone isolated from *A. obtusus* [154] presented not only anti-inflammatory and anti-oxidant activities, but also anti-apoptotic and anti-bacterial effects against *Helicobacter pylori*. The inhibitory effects of 1,7-dihydroxy-8-methoxyxanthone (**97**), cochinchinone A (**98**), formoxanthone A (**99**), macruraxanthone (**4**), cochinxanthone E (**100**), pruniflorone L (**101**), dulcisxanthone F (**102**), 5,9-dihydroxy-8-methoxy-2,2-dimethyl-7-(3-methyl-but-2-enyl)-2*H*,6*H*-pyrano-[3,2*b*]-xanthone (**103**), pruniflorone K (**104**), and garcinone B (**105**) (Figure 9) obtained from *C. formosum* ssp. *pruniflorum* showed inhibitory effects on NO production by murine macrophage-like RAW264.7 cells [41]. Among them, compounds **99**, **102**, **103** displayed good suppressive ability on NO production with IC_50_ values of 8.0, 3.9, and 4.3 μM, respectively, while **98**, **100** and **105** showed moderate activity with IC_50_ values of 12.6, 12.8 and 11.8 μM, respectively. The investigation indicated that tetraoxygenated xanthone skeleton exhibited inhibition of NO production greater than trioxygenated xanthone skeleton, while the methoxyl group at C-3 or C-7 on the tetraoxygenated isoprenylated-xanthone skeleton was an essential group.

## 4. Conclusions

As a class of secondary metabolites obtained from a number of herbal medicines, xanthones are playing more and more important roles in new drug research and development. The pharmacokinetics and toxicity (PK/tox) properties of xanthones, as part of the most crucial preclinical studies, have proved that xanthones are promising drug candidates owing to their high efficacy and low toxicity [186,187].

In this paper, we have summarized the plant sources, bioactivity and the SARs of xanthones from literature published over the last few decades. As a result, 168 species of herbal medicine plants belonging to 58 genera, and 24 families were found to be enriched in xanthones. Among them, the *Calophyllum*, *Cratoxylum*, *Cudrania*, *Garcinia*, *Gentiana*, *Hypericum* and *Swertia* genera are the plant resource with the most development prospect. Xanthones display multiple bioactivities, which may be useful for new drug development for cancer, inflammation, bacterial, fungal and viral infection, diabetes, and so on.

## Figures and Tables

**Table 1:** Plant distribution of xanthones.

Family	Genus	Species	Reference
Acanthaceae	*Andrographis*	*A. paniculata* (Burm. f.) Nees	[11]
Anacardiaceae	*Mangifera*	*M. indica* L.	[12]
	*Rhus*	*R. coriaria* L.	[13]
Annonaceae	*Anaxagorea*	*A. luzonensis* A. Gray	[14]
	*Guatteria*	*G. blepharophylla* Mart.	[15]
Asparagus	*Ledebouria*	*L. ovatifolia* (Schrad.) Jessop	[16]
Asparagaceae	*Anemarrhena*	*A. asphodeloides* Bunge	[17]
	*Drimiopsis*	*D. maculate* Lindl. & Paxton	[18]
Asteraceae	*Santolina*	*S. insularis* (Gennari ex Fiori) Arrigoni	[19]
Bignoniaceae	*Arrabidaea*	*A. samydoides* (Cham.) Sandwith	[20]
Bombacaceae	*Bombax*	*B. ceiba* L.	[21]
Clusiaceae (or Guttiferae)	*Allanblackia*	*A. floribunda* Oliv.	[22]
		*A. gabonensis* (Pellegr.) Bamps	[23]
		*A. monticola* Staner L. C.	[24]
	*Bonnetia*	*B. stricta* Mart.	[25]
	*Calophyllum*	*C. brasiliense* Cambess.	[26]
		*C. caledonicum* Vieill. ex Planch. & Triana	[27]
		*C. decipiens* Wight	[28]
		*C. gracilipes* Merr.	[29]
		*C. inophyllum* L.	[30]
		*C. membranaceum* Gardner & Champ.	[31]
		*C. panciflorum* A. C. Smith	[32]
		*C. pinetorum* Bisse	[33]
		*C. soulattri* Burm. f.	[34]
		*C. symingtonianum* M.R. Hend. & Wyatt-Sm.	[35]
		*C. thorelii* Pierre	[36]
		*C. thwaitesii* Planch. & Triana	[37]
	*Chrysochlamys*	*C. tenuis* Hammel	[38]
	*Clusia*	*C. pernambucensis* G. Mariz	[39]
	*Cratoxylum*	*C. cochinchinensis* (Lour.) Blume	[40]
		*C. formosum* sp. *Pruniflorum* (Kurz) Gogelein	[41]
	*Garcinia*	*G. afzelii* Engl.	[42]
		*G. bracteata* C.Y. Wu ex Y.H. Li	[43]
		*G. cambogia* (Gaertn.) Desr.	[44]
		*G. cantleyana* Whitmore	[45]
		*G. cowa* Roxb. ex Choisy	[46]
		*G. dioica* Blume	[47]
		*G. dulcis* (Roxb.) Kurz	[48]
		*G. eugenifolia* Wall. ex T. Anderson	[49]
		*G. fusca* Pierre	[50]
		*G. goudotiana* (Planch. & Triana) P. Sweeney & Z.S. Rogers	[51]
		*G. griffthii* T. Anderson	[52]
		*G. hanburyi* Hook. f.	[53]
		*G. hombroniana* Pierre	[54]
		*G. lancilimba* C.Y. Wu ex Y.H. Li	[55]
		*G. lateriflora* Blume	[56]
		*G. linii* C.E. Chang	[57]
		*G. mangostana* L.	[58]
		*G. merguensis* Wight	[59]
		*G. multiflora* Champ. ex Benth.	[60]
		*G. nigrolineata* Planch. ex T. Anderson	[61]
		*G. nitida* Pierre	[62]
		*G. nobilis* Engl.	[63]
		*G. nujiangensis* C.Y. Wu & Y.H. Li	[64]
		*G. oblongifolia* Champ. ex Benth.	[65]
		*G. oligantha* Merr.	[66]
		*G. oliveri* Pierre	[67]
		*G. parvifolia* (Miq.) Miq.	[68]
		*G. paucinervis* Chun & F.C. How	[69]
		*G. pedunculata* Roxb. ex Buch.-Ham.	[70]
		*G. penangiana* Pierre	[71]
		*G. polyantha* Oliv.	[72]
		*G. porrecta* Laness.	[68]
		*G. propinqua* Craib	[73]
		*G. rigida* Miq.	[74]
		*G. schomburgkiana* Pierre	[75]
		*G. scortechinii* King	[76]
		*G. smeathmannii* (Planch. & Triana) Oliv.	[77]
		*G. staudtii* Engl.	[78]
		*G. subelliptica* Merr.	[79]
		*G. succifolia* Kurz	[80]
		*G. tetralata* C.Y. Wu ex Y.H. Li	[81]
		*G. vieillardii* Pierre	[82]
		*G. virgate* Vieill.	[83]
		*G. xanthochymus* Hook. f. ex T. Anderson	[84]
		*G. xipshuanbannaensis* Y.H. Li	[85]
	*Kielmeyera*	*K. coriacea* Mart.	[86]
		*K. variabilis* Mart. & Zucc.	[87]
	*Mammea*	*M. siamensis* T. Anderson	[88]
	*Mesua*	*M. ferrea* L.	[89]
		*M. hexapetala* (Hook. f.) P.S. Ashton	[90]
	*Psorospermum*	*P. adamauense* Engl.	[91]
		*P. febrifugum* Spach	[92]
		*P. molluscum* (Pers.) Hochr.	[93]
	*Rheedia*	*R. acuminata* (Ruiz & Pav.) Planch. & Triana	[94]
	*Symphonia*	*S. globulifera* L.f.	[95]
	*Vismia*	*V. laurentii* De Wild.	[96]
		*V. rubescens* Oliv.	[96]
Fabaceae	*Caesalpinia*	*C. sappan* L.	[97]
	*Cassia*	*C. obtusifolia* L.	[98]
	*Cyclopia*	*C. genistoides* (L.) Vent.	[99]
	*Desmodium*	*D. caudatum* (Thunb.) DC.	[100]
Ganodermataceae	*Gyrophora*	*G. proboscidea* (L.) Ach.	[101]
Gentianaceae	*Centaurium*	*C. spicatum* (L.) Fritsch	[102]
	*Comastoma*	*C. pedunculatum* (Royle ex G. Don) Holub	[103]
		*C. pulmonarium* (Turcz.) Toyok.	[104]
	*Gentiana*	*G. dinarica* Beck	[105]
		*G. kochiana* Perr. & Songeon	[106]
		*G. lutea* L.	[107]
		*G. tizuensis* Franch.	[108]
		*G. utriculosa* L.	[109]
		*Gentiana dinarica* Beck.	[110]
	*Gentianella*	*G. acuta* (Michx.) Hiitonen	[111]
		*G. amarella* (L.) Harry Sm.	[112]
		*G. turkestanorum* (Gand.) Holub	[113]
	*Gentianopsis*	*G. barbata* (Froel.) Ma	[114]
		*G. paludosa* (Hook. f.) Ma	[115]
	*Halenia*	*H. corniculata* (L.) Cornaz	[116]
		*H. elliptica* D. Don	[117]
	*Lomatogonium*	*L. carinthiacum* (Wulfen) A. Braun	[118]
	*Schultesia*	*S. lisianthoides* (Griseb.) Benth. & Hook. f. ex Hemsl.	[119]
	*Swertia*	*S. chirayita* (Roxb.) H. Karsten	[120]
		*S. cordata* (Wall. ex G. Don) C.B. Clarke	[121]
		*S. corymbosa* Wight ex Griseb.	[122]
		*S. cuneata* Wall. ex D. Don	[123]
		*S. elata* Harry Sm.	[124]
		*S. franchetiana* Harry Sm.	[125]
		*S. kouitchensis* Franch.	[126]
		*S. longifolia* Boiss.	[127]
		*S. minor* (Griscb.) Knobl.	[128]
		*S. mussotii* Franch.	[129]
		*S. paniculata*	[130]
		*S. pseudochinensis* H. Hara	[131]
		*S. punicea* Hemsl.	[132]
		*S. speciosa* Wall.	[133]
	*Tachia*	*T. grandiflora* Maguire & Weaver	[134]
Hippocrateaceae	*Salacia*	*S. chinensis* L.	[135]
		*S. elliptica* (Mart.) G. Don	[136]
Hyacinthaceae	*Scilla*	*S. scilloides* (Lindl.) Druce	[137]
Hypericaceae	*Hypericum*	*H. ascyron* L.	[138]
		*H. attenuatum* Fisch. ex Choisy	[139]
		*H. chinense* L.	[140]
		*H. erectum* Thunb.	[141]
		*H. lanceolatum* Lam.	[142]
		*H. oblongifolium* Choisy	[143]
		*H. patulum* Thunb.	[144]
		*H. perforatum* L.	[145]
		*H. sampsonii* Hance	[146]
		*H. scabrum* L.	[147]
		*H. styphelioides* A. Rich.	[148]
Iridaceae	*Iris*	*I. nigricans* Dinsm.	[149]
Loganiaceae	*Anthocleista*	*A. schweinfurthii* Gilg	[150]
		*A. vogelii* Planch.	[151]
Moraceae	*Artocarpus*	*A. kemando* Miq.	[152]
		*A. nobilis* Thwaites	[153]
		*A. obtusus* F.M. Jarrett	[154]
	*Cudrania*	*C. cochinchinensis* (Lour.) Yakuro Kudo & Masam.	[155]
		*C. fruticosa* (Roxb.) Wight ex Kurz	[156]
		*C. tricuspidata* (Carrière) Bureau ex Lavallée	[157]
	*Maclura*	*M. cochinchinensis* (Lour.) Corner	[158]
Onagraceae	*Oenothera*	*O. biennis* L.	[159]
Parmeliaceae	*Usnea*	*U. hirta* (L.) Weber ex F.H. Wigg	[160]
Polygalaceae	*Bredemeyera*	*B. floribunda* Willd.	[161]
	*Moutabea*	*M. guianensis* Aubl.	[162]
	*Polygala*	*P. caudata* Rehder & E.H. Wilson	[163]
		*P. crotalarioides* Buch.-Ham. ex DC.	[164]
		*P. cyparissias* A. St.-Hil. & Moq.	[165]
	*Securidaca*	*P. hongkongensis* Hemsl.	[166]
		*P. japonica* Houtt.	[167]
		*P. karensium* Kurz	[168]
		*P. tenuifolia* Willd.	[169]
		*P. wattersii* Hance	[170]
		*S. inappendiculata* Hassk.	[171]
		*S. longepedunculata* Fresen.	[172]
Rubiaceae	*Coffea*	*C. pseudozanguebariae* Bridson	[173]
	*Morinda*	*M. citrifolia* L.	[174]
Theaceae	*Pentadesma*	*P. butyrace* Sabine	[175]
Xanthorrhoeaceae	*Bulbine*	*B. frutescens* (L.) Willd.	[176]
Zingiberaceae	*Hedychium*	*H. gardnerianum* Sheppard ex Ker Gawl.	[177]
